# Impact of postoperative complications on the colorectal cancer survival and recurrence: analyses of pooled individual patients’ data from three large phase III randomized trials

**DOI:** 10.1002/cam4.1126

**Published:** 2017-06-22

**Authors:** Toru Aoyama, Koji Oba, Michitaka Honda, Sotaro Sadahiro, Chikuma Hamada, Shuhei Mayanagi, Mitsuro Kanda, Hiromichi Maeda, Kosuke Kashiwabara, Junichi Sakamoto, Shigetoyo Saji, Takaki Yoshikawa

**Affiliations:** ^1^ Department of Surgery Yokohama City University Yokohama Japan; ^2^ Department of Biostatistics The University of Tokyo Tokyo Japan; ^3^ Department of Minimally Invasive Surgical and Medical Oncology Fukushima Medical University, Fukushima Japan; ^4^ Department of Surgery Tokai University Isehara Japan; ^5^ Faculty of Engineering Tokyo University of Science Tokyo Japan; ^6^ Department of Surgery Keio University School of Medicine Tokyo Japan; ^7^ Department of Gastroenterological Surgery Nagoya University Graduate School of Medicine Nagoya Japan; ^8^ Cancer Treatment Center Kochi Medical School Hospital Kochi Japan; ^9^ Tokai Central Hospital Kakamigahara Japan; ^10^ Japanese Foundation for Multidisciplinary Treatment of Cancer Tokyo Japan; ^11^ Department of Gastrointestinal Surgery Kanagawa Cancer Center Yokohama Japan

**Keywords:** Colorectal cancer, recurrence, surgical complication, survival

## Abstract

This study assessed the impact of postoperative complications on the colorectal cancer survival and recurrence after curative surgery using pooled individual patients’ data from three large phase III randomized trials. In total, 5530 patients were included in this study. The patients were classified as those with postoperative complications (C group) and those without postoperative complications (NC group). The risk factors for the overall survival (OS) and the disease‐free survival (DFS) were analyzed. Postoperative complications were found in 861 (15.6%) of the 5530 patients. The OS and DFS rates at 5 years after surgery were 68.9% and 74.8%, respectively, in the C group and 75.8% and 82.2%, respectively, in the NC group, values that were significantly different between the two groups (*P* < 0.001). The multivariate analysis demonstrated that postoperative complications were a significant independent risk factor for the OS and DFS. Postoperative complications can worsen the colorectal cancer survival and risk of recurrence. Surgical morbidity must be considered as a stratification factor in future phase III trials evaluating the effects of adjuvant chemotherapy on colorectal cancer.

## Introduction

Colorectal cancer is the third most commonly diagnosed cancer in males and the second in females, with an estimated 1.4 million new cases and 693,900 deaths occurring in 2012 1. Complete resection is essential for achieving a cure. Although the resection rate has gradually increased, some patients still experience recurrence, even after curative surgery 2. Once recurrence has developed, the prognosis is poor. Therefore, it is important to identify reliable predictive factors for patients at high risk of recurrence.

Postoperative complications are observed in around 20% of patients who receive curative surgery with lymph node dissection for colorectal cancer 3, 4, 5, 6. Recent studies have shown that the postoperative complications were risk factors for the survival or disease recurrence in various types of malignancies 7, 8, 9, 10, 11, 12. Furthermore, some authors have shown that the immunological response against postoperative complications enhanced the viability of undetectable residual tumor cells after surgery, thereby increasing the risk of disease recurrence 13, 14. However, despite numerous studies performed in patients with various types of malignancies, most previous studies have used and evaluated retrospectively collected data with relatively small sample sizes of less than 200 from a single institution. Retrospective studies have many limitations, such as unspecified indications of surgery, heterogeneous populations, heterogeneous treatments, and description bias of surgical morbidity. Physicians can sometimes hesitate to perform postoperative chemotherapy in frail patients who easily develop surgical morbidity. Such frail patients might therefore have a particularly poor prognosis. To overcome such limitations associated with retrospective studies, we focused on cases that were enrolled in large randomized clinical trials of adjuvant chemotherapy by pooling individual patients’ data. The impact of such morbidities on the prognosis must be reevaluated in a large cohort with prespecified indications of surgery, predefined treatments, and prospectively collected data from clinical trials.

The aim of this study was to determine whether or not the overall survival (OS) and disease‐free survival (DFS) were affected by the development of postoperative complications in patients who underwent curative resection for colorectal cancer by a pooled analysis of three large phase III studies performed in Japan. This study had the ultimate goal of evaluating and confirming the actual impact of surgical morbidity on the prognosis of colorectal cancer patients who received curative surgery.

## Patients and Methods

### Patients

The data and outcomes or patients enrolled in three phase III trials of Japanese Foundation for Multidisciplinary Treatment of Cancer (JFMC) studies (7, 15, and 33) were pooled 15, 16, 17.

### JFMC trials in the pooled analysis

#### JFMC 7 and 15

These two randomized trials were relatively large‐scale trials conducted in Japan. They focused on the long‐term utilization of oral 5‐fluorouracil (FU) as adjuvant chemotherapy for colon or rectal cancer and compared the overall survival in the surgery‐alone arm with the treatment arms. The basic study designs of these trials were very similar, and included the following key eligibility criteria: stage of cancer macroscopic Dukes’ B and C, age <75 years, no severe complications, and follow‐up period 5 years. The main adjuvant chemotherapy was 1‐year administration of oral 5‐FUs (JFMC7‐1: 200 mg/day 5‐FU; JFMC7‐2 and JFMC15: 300 mg/day 1‐hexycarbamoyl‐5‐fluorouracil [carmofur, HCFU]). HCFU is an oral fluorinated pyrimidine developed as a 5‐FU lipophilic masked compound. All eligible patients were the target analysis set for the statistical analysis. There were 3394 patients enrolled in total for the JFMC 7 trial between February 1986 and December 1988 and 2315 enrolled in total in total for JFMC 15 trial between January 1989 and December 1990. The details of the protocol and primary analyses of JFMC 7 and JFMC 15 have been reported previously.

#### JFMC 33

This phase III trial randomly assigned 1071 eligible patients from 2005 to 2007 at 233 centers to receive tegafur (UFT, 300 mg/m^2^/day as tegafur)/leucovorin (LV, 75 mg/day) for 28 of 35 days for 6 months in the control group or for 5 consecutive days per week for 18 months. Patients with curatively resected stage IIB/III colon cancer were eligible for enrollment in this trial. The primary endpoint was the overall survival. The details and primary analyses of JFMC 33 have been reported previously.

In this pooled analysis, the eligible patients were those who had stage I/II/III colorectal cancer and had received over D2 lymph node dissection. Thus, 754 and 496 patients were excluded from the intention‐to‐treat cohort of each study. In total, 5530 patients were included in the present study. Individual patient data were obtained and analyzed for the study.

### Postoperative complications

Each study prespecified that physicians must report any surgical morbidity at study enrollment. All information on complications was extracted from the case report forms of each trial. Major surgical complications were defined as follows: anastomotic leakage, pneumonia, bowel obstruction/ileus, surgical site infection, postoperative bleeding, urinary tract infection, and fistula. The patients were classified as those with postoperative complications (C group) and without postoperative complications (NC group).

### Follow‐up

Patients were followed up in accordance with the protocol of each study. Briefly, during protocol treatment, the clinical findings and laboratory data were evaluated every 2 weeks. After completion of the protocol treatment, patients were followed up in accordance with a predefined surveillance schedule until recurrence or death was confirmed for 5 years after surgery. Recurrence was assessed based on computed tomography (CT) scans. These tests were carried out every 4 months during the first 2 years after surgery and once every 6 months from the third year onward.

### Evaluations and statistical analyses

The background characteristics of the postoperative clinical and pathological parameters between the C and NC groups were compared using the Mann–Whitney test or chi‐square test. The OS was defined as the period between surgery and any cause of death. The DFS was defined as the period between surgery and the occurrence of recurrence, second cancer, or death, whichever came first. The data for patients who had not experienced an event were censored at the date of the final observation. The OS and DFS curves were calculated using the Kaplan–Meier method, and were compared by the log‐rank test. A Cox proportional hazards model was used to perform the univariate and multivariate survival analyses. In the multivariate analysis, adjuvant chemotherapy, age (<60 or ≥60), gender, tumor diameter (<50 or ≥50 mm), tumor location (colon or rectum), T status (T1 to T3 or T4), lymph node metastases (positive or negative), and lymphadenectomy (D2 or D3) were considered as available confounders based on a prior knowledge. Trials (JFMC7, 15, and 33) were also considered as a fixed effect in all multivariate Cox regression model. A *P* < 0.05 (two‐sided) was defined as being statistically significant. SAS version 9.4 (SAS Institute, Inc., Cary, NC) was used for all statistical analyses. This study was approved by the ethical committee of the Japanese Foundation for Multidisciplinary Treatment of Cancer.

## Results

### Patients

The data of 5530 individual patients were evaluated in this study. The patients’ ages ranged from 20 to 75 years (median: 60 years); 3104 patients were males and 2426 were females. The median follow‐up period was 5 years. Patients’ demographics and clinical characteristics are summarized in Table**** 1. The age, gender, sex, tumor location, tumor diameter, UICC T status, and UICC N status were significantly different between the two groups. The incidence of rectal cancer tended to be higher in the C group than in the NC group.

**Table 1:** A comparison of the clinicopathological factors between the patients with and without surgical complications

Factors	All cases (*n* = 5530)		C group (*n* = 861)		NC group (*n* = 4669)		*P*
	*N*	%	*N*	%	*N*	%	
Gender							<0.001
Male	3104	56.1	583	67.7	2521	54.0	
Female	2426	43.9	278	32.3	2148	46.0	
Age (years)							<0.001
Median (range)	60 (20–75)		59 (27–75)		61 (20–75)		
Tumor location							<0.001
Colon	4036	73.0	420	48.8	3616	77.4	
Rectal	1492	27.0	441	51.2	1051	22.5	
Diameter of tumor (mm)							0.002
Median (range)	50 (10–280)		50 (10–140)		50 (10–280)		
Histology							0.048
Well‐mod	5199	94.0	821	95.4	4378	93.8	
Poor	136	2.5	11	1.3	125	2.7	
Others	195	3.5	29	3.4	166	3.6	
UICC T status							<0.001
T1–T3	3767	68.1	536	62.3	3231	69.2	
T4	1763	31.9	325	37.7	1438	30.8	
LN node metastases							<0.001
Negative	3146	56.9	543	63.1	2603	55.8	
Positive	2362	42.7	314	36.5	2048	43.9	
Missing	22	0.4	4	0.5	18	0.4	
Lymph node dissection							0.111
D2	2293	41.5	378	43.9	1915	41.0	
D3	3227	58.4	481	55.9	2746	58.8	
Missing	10	0.1	2	0.2	8	0.2	
Adjuvant chemotherapy							0.246
Yes	3609	65.3	547	63.5	3062	65.6	
No	1921	34.7	314	36.5	1607	34.4	

Well‐mod, well to moderately differentiated; Poor, poorly differentiated; UICC, Union for International Cancer Control; LN, lymph node; C group, patients with surgical complications, NC group, patients without surgical complications.

### Surgical morbidity and mortality

Postoperative complications were found in 861 (15.6%) of the 5530 patients. The details of the complications are shown in Table 2. Bowel obstruction/ileus was the most frequently observed complication, followed by wound abscess and anastomotic leakage.

**Table 2:** Details of postoperative complications

	Number of patients	%
Bowel obstruction/ileus	316	5.7
Surgical site infection	288	5.2
Anastomotic leakage	170	3.1
Urinary tract infection	119	2.2
Fistula	47	0.8
Pneumonia	42	0.8
Postoperative bleeding	38	0.7

### Survival analysis

**Figure 1:**
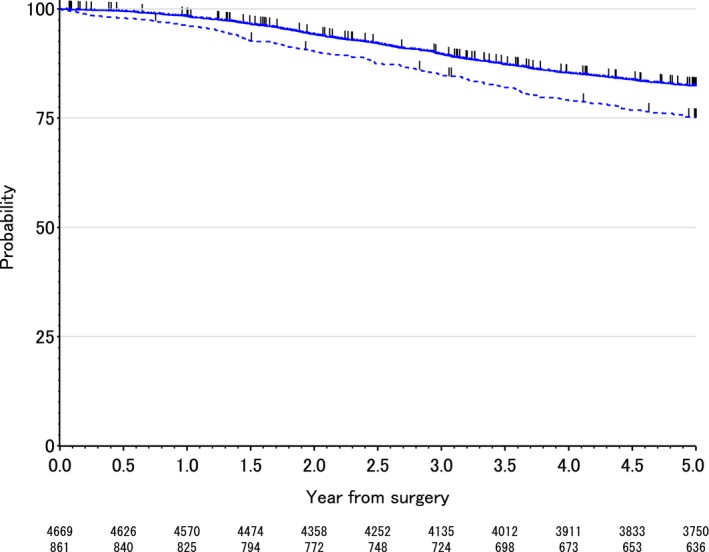
The overall survival curves of the C group (those with surgical complications) and NC group (those without surgical complications).

The OS rates at 3 and 5 years after surgery were 84.6% and 74.8% in the C group and 89.5% and 82.2% in the NC group, respectively; these values were significantly different between the two groups (*P* < 0.0001). The OS curves are shown in Figure 1. In the univariate analysis, postoperative complication, gender, tumor diameter, tumor location, the T status, N status, lymphadenectomy, and use of adjuvant chemotherapy were all found to be significantly associated with the OS. The multivariate analysis showed that postoperative complications increased the risk of death by 31% (adjusted hazard ratio = 1.31; 95% confidence interval, 1.12–1.54; *P* = 0.001; Table 3).

**Table 3:** Univariate and multivariate Cox proportional hazards analyses of the clinicopathological factors for the disease‐free survival

Factors	Number	Univariate analysis			Multivariate analysis^a^		
		HR	95% CI	*P*	HR	95% CI	*P*
Surgical complication				<0.001			0.003
No	4669	1.00			1.00		
Yes	861	1.35	1.18–1.55		1.24	1.08–1.42	
Age (years)				0.115			0.093
<60	2571	1.00			1.00		
≥60	2959	1.09	0.98–1.21		1.10	0.99–1.22	
Gender				0.001			0.002
Female	2420	1.00			1.00		
Male	3104	1.24	1.11–1.38		1.19	1.07–1.33	
Tumor diameter (mm)				0.041			0.088
<50	2423	1.00			1.00		
≥50	3095	1.12	1.01–1.25		1.10	0.99–1.23	
Tumor location				<0.001			<0.001
Colon	4036	1.00			1.00		
Rectal	1492	1.48	1.33–1.66		1.37	1.21–1.55	
UICC T status				<0.001			<0.001
T1–T3	3767	1.00			1.00		
T4	1763	1.72	1.54–1.91		1.55	1.38–1.73	
Lymph node metastases				<0.001			<0.001
Negative	3146	1.00			1.00		
Positive	2362	2.15	1.93–2.39		2.38	2.12–2.66	
Lymph node dissection				0.002			0.001
D3	3227	1.00			1.00		
D2	2293	1.18	1.06–1.31		1.19	1.07–1.33	
Adjuvant chemotherapy				0.005			0.023
No	1921	1.00			1.00		
Yes	3609	0.84	0.74–0.95		0.87	0.77–0.98	

UICC, Union for International Cancer Control; HR, hazard ratio; CI, confidence interval.

a The study was adjusted as a fixed effect in the multivariate Cox model.

**Figure 2:**
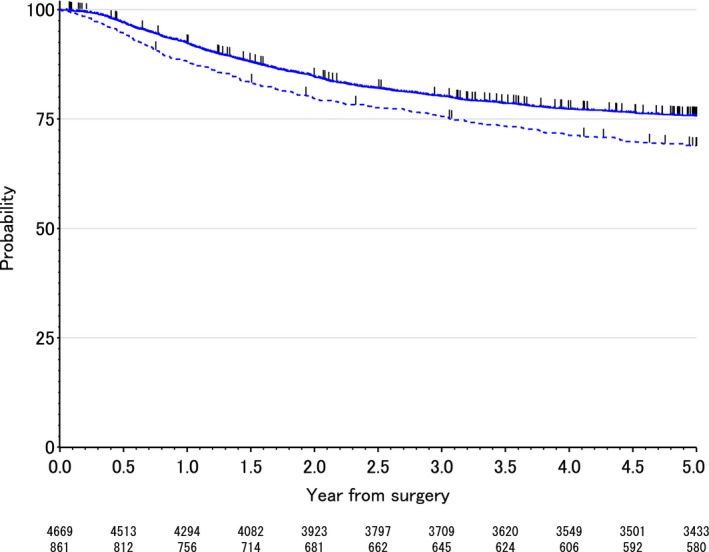
The disease‐free survival curves of the C group (those with surgical complications) and NC group (those without surgical complications).

The DFS rates at 3 and 5 years after surgery were 75.6% and 68.9% in the C group patients and 80.2% and 75.8% in the NC group patients, respectively. The DFS was therefore significantly worse in the C group than in the NC group (*P* < 0.001). The DFS curves are shown in Figure 2. In the univariate analysis, postoperative complication, gender, tumor diameter, tumor location, the T status, N status, lymphadenectomy, and use of adjuvant chemotherapy were found to be significantly associated with the DFS. The multivariate analysis showed that postoperative complications were significant independent risk factors for the DFS (Table 4). Adjusted hazard ratio for DFS was 1.24 (95% confidence interval, 1.08–1.42; *P* = 0.003).

**Table 4:** Univariate and multivariate Cox proportional hazard analyses of the clinicopathological factors for the overall survival

Factors	Number	Univariate analysis			Multivariate analysis^a^		
		HR	95% CI	*P*	HR	95% CI	*P*
Surgical complication				<0.001			0.001
No	4669	1.00			1.00		
Yes	861	1.49	1.28–1.73		1.31	1.12–1.54	
Age (years)				0.039			0.003
<60	2571	1.00			1.00		
≥60	2959	1.14	1.01–1.29		1.21	1.07–1.37	
Gender				0.289			0.034
Female	2420	1.00			1.00		
Male	3104	1.23	1.09–1.40		1.15	1.01–1.30	
Tumor diameter (mm)				<0.001			0.005
<50	2423	1.00			1.00		
≥50	3095	1.25	1.11–1.42		1.20	1.06–1.37	
Tumor location				<0.001			0.001
Colon	4036	1.00			1.00		
Rectal	1492	1.56	1.38–1.78		1.27	1.11–1.47	
UICC T status				<0.001			<0.001
T1–T3	3767	1.00			1.00		
T4	1763	1.71	1.52–1.94		1.53	1.34–1.73	
Lymph node metastases				<0.001			<0.001
Negative	3146	1.00			1.00		
Positive	2362	1.98	1.75–2.24		2.48	2.17–2.83	
Lymph node dissection				0.002			0.007
D3	3227	1.00			1.00		
D2	2293	1.22	1.08–1.38		1.19	1.05–1.35	
Adjuvant chemotherapy				0.065			0.231
No	1921	1.00			1.00		
Yes	3609	0.90	0.81–1.01		0.92	0.80–1.06	

UICC, Union for International Cancer Control, HR, hazard ratio, CI, confidence interval.

a The study was adjusted as a fixed effect in the multivariate Cox model.

### Subgroup analysis

The subgroup analyses were performed according to the type of treatment after surgery. The 5‐year OS rates in patients receiving adjuvant chemotherapy or surgery alone were 77.4% and 71.4% in the C group patients, respectively, and were 83.2% and 81.0% in the NC group patients, respectively (Fig. S1). In patients randomized to surgery‐alone group, adjusted hazard ratio of postoperative complications for OS and DFS was 1.44 (95% confidence interval, 1.13–1.84) and 1.32 (95% confidence interval, 1.06–1.64), respectively. In patients randomized to the adjuvant chemotherapy group, adjusted hazard ratio of postoperative complications for OS and DFS was 1.23 (95% confidence interval, 1.00–1.52) and 1.19 (95% confidence interval, 0.99–1.43), respectively.

Impacts stratified by the site of the primary tumor were also examined. Crude Kaplan–Meier curves in colon cancer and rectum cancer were shown in Figure S2. In colon cancer patients, adjusted hazard ratio of postoperative complications for OS and DFS was 1.30 (95% confidence interval, 1.03–1.64) and 1.28 (95% confidence interval, 1.05–1.57), respectively. In rectum cancer patients, adjusted hazard ratio of postoperative complications for OS and DFS was 1.31 (95% confidence interval, 1.05–1.63) and 1.19 (95% confidence interval, 0.98–1.44), respectively.

## Discussion

This study examined whether or not postoperative complications were associated with a poorer OS and DFS after curative surgery for colorectal cancer in combined analyses of individual patients’ data from the three large phase III studies evaluating the effects of adjuvant treatment. Our findings clearly indicated that postoperative complications were a significant independent risk factor for the OS and DFS. Similar trends were observed, regardless of the adjuvant treatment or tumor location.

The present study only included patients enrolled in phase III studies of adjuvant chemotherapy, meaning that all patients who had developed surgical morbidities had completely recovered from their complications before study entry and satisfied the eligibility criteria of each study. All patients were considered to be ready to receive adjuvant chemotherapy. Previous studies were associated with the major criticism that frail patients, who easily develop surgical complications, have a worse prognosis than nonfrail patients because of their frailty and not because of adjuvant chemotherapy. The present study clearly showed that surgical complications were associated with a worse prognosis, with no relation to the addition of adjuvant chemotherapy or type of adjuvant chemotherapy. This finding strongly suggested that the negative survival impact was due not to frail patients who did not receive adjuvant chemotherapy but to surgical morbidity itself.

In the present study, adjusted hazard ratio for OS was 1.31 (95% confidence interval, 1.12–1.54) and that for DFS was 1.24 (95% confidence interval, 1.08–1.42). Similar hazard ratio and 95% confidence interval were observed in the previous largest observational studies. Law et al. examined 1657 colorectal cancer patients 13 and found that postoperative complications were an independent factor associated with a worse overall survival (hazard ratio of 1.26) and a higher rate of overall recurrence (hazard ratio of 1.26). Similar hazard ratios were also reported in other studies 18.

Although the present results are considered to be solid in terms of the study design and sample size, several limitations warrant mention. First, the patients in this cohort met the strict inclusion criteria of each clinical trial, which may have contained selection bias. When considering the generalizability, the impact of complications may be emphasized in this cohort. Second, there is a time bias in this study, as the data were collected over the relatively long period of 1986–2007. Surgical procedures and perioperative care might have changed over the years. Third, the definition and severity of morbidity were not strictly defined in each phase III study. The postoperative surgical complications were reported based not on the study protocol but on the judgment of individual physicians. Although the incidence of morbidity in this study was similar to that in other large studies 19, 20, some surgical complications might have been underestimated.

A further important limitation of all of the available data regarding postoperative complications, including the current study, is the lack of consensus regarding the appropriate cut‐off point and definition for evaluation of the postoperative complications. In this study, the definition and severity of the postoperative surgical complications were not determined in the three phase III studies. The postoperative surgical complications of this study were reported by the individual physicians, not based on a specific protocol. Although the incidence of the postoperative surgical complications was similar to other large phase III trials, some postoperative surgical complications might be underestimated. This would also greatly aid the use of the postoperative complications as stratification factor in future clinical trials.

In conclusion, this study confirmed that the development of postoperative complications was a risk factor for the overall survival and disease recurrence in patients who underwent curative surgery for colorectal cancer. Surgical morbidity must be considered as a stratification factor in future phase III trials evaluating the effects of adjuvant chemotherapy for colorectal cancer.

## Conflict of Interest

None declared.

## Supporting information


**Figure S1.** The overall survival curves of the C group (those with surgical complications) and NC group (those without surgical complications) according to the type of treatment after surgery.

**Figure S2.** The overall survival curves of the C group (those with surgical complications) and NC group (those without surgical complications) according to the primary tumor location.
