# Supplementary figures and images for: Impact of postoperative complications on the colorectal cancer survival and recurrence: analyses of pooled individual patients’ data from three large phase III randomized trials
Source: Cancer Med. 2017 Jun 22;6(7):1573–80. doi: 10.1002/cam4.1126 (PMC5504309; doi:10.1002/cam4.1126)

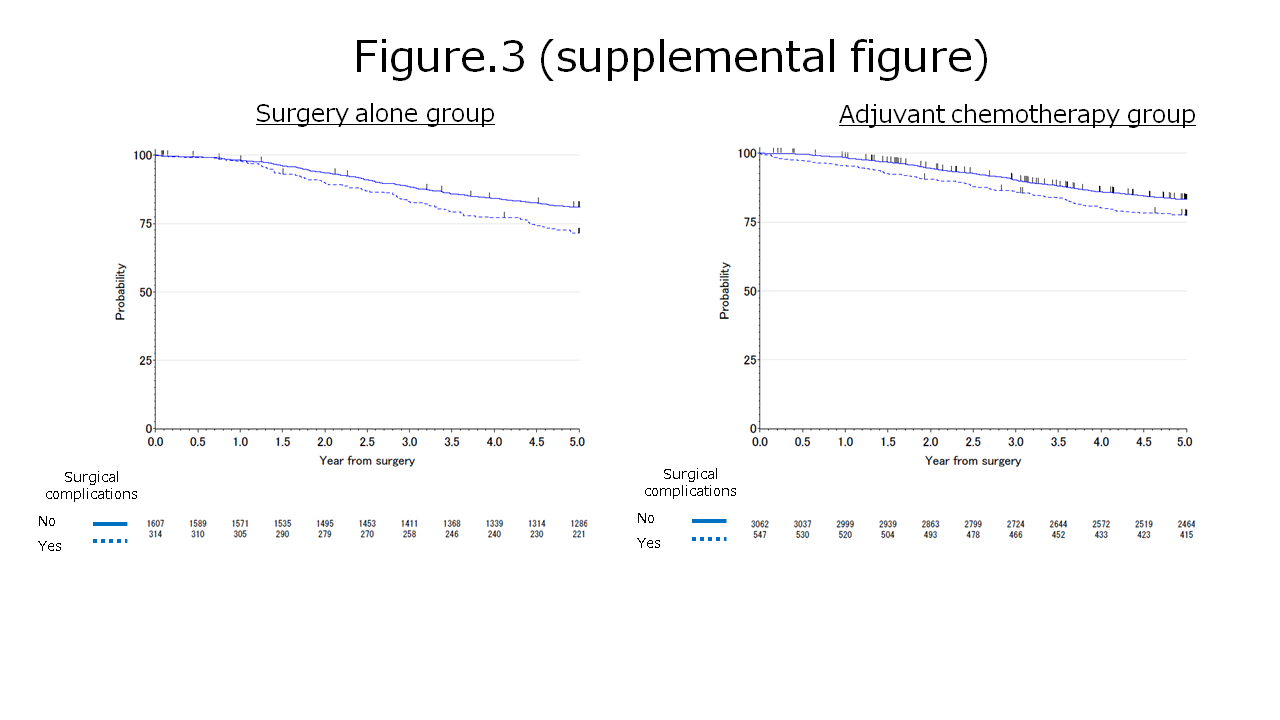

Supplement: Supplementary file 1 — Figure S1. The overall survival curves of the C group (those with surgical complications) and NC group (those without surgical complications) according to the type of treatment after surgery. [file CAM4-6-1573-s001.TIF]

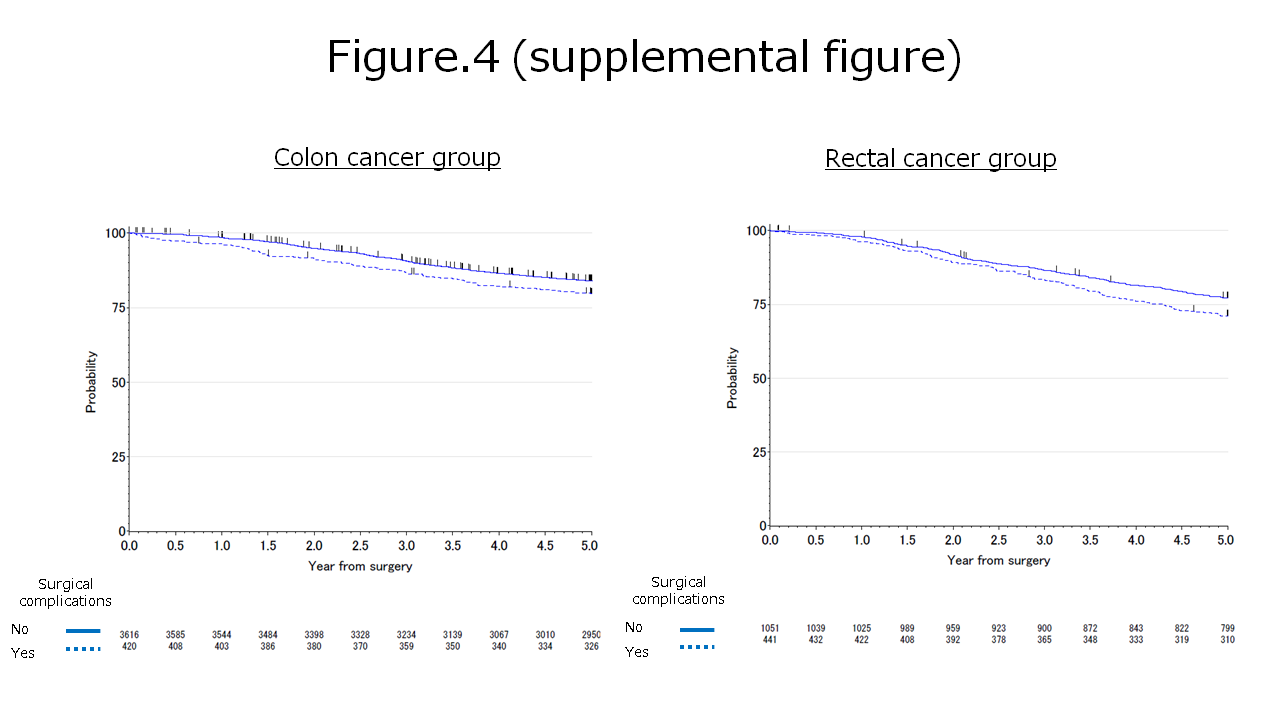

Supplement: Supplementary file 2 — Figure S2. The overall survival curves of the C group (those with surgical complications) and NC group (those without surgical complications) according to the primary tumor location. [file CAM4-6-1573-s002.TIF]
